# Green fabrication of hierarchical zeolites from natural minerals

**DOI:** 10.1093/nsr/nwaa197

**Published:** 2020-08-31

**Authors:** Yuanyuan Yue, Haibo Zhu, Tinghai Wang, Xiaojun Bao

**Affiliations:** National Engineering Research Center of Chemical Fertilizer Catalyst, College of Chemical Engineering, Fuzhou University, China; National Engineering Research Center of Chemical Fertilizer Catalyst, College of Chemical Engineering, Fuzhou University, China; National Engineering Research Center of Chemical Fertilizer Catalyst, College of Chemical Engineering, Fuzhou University, China; National Engineering Research Center of Chemical Fertilizer Catalyst, College of Chemical Engineering, Fuzhou University, China

## TRADITIONAL PROCESS FOR SYNTHESISING HIERARCHICAL ZEOLITES

Zeolites are considered a cornerstone of the modern petroleum and chemical industries, because their human-made synthesis and industrial applications have greatly helped drive the revolutions in petroleum refining and chemical production processes. However, it is widely accepted that the intracrystalline diffusion limitation, which arises from the relatively small micropores of zeolites (<1 nm), substantially limits the performance of zeolite-based catalysts [1]. In particular, when zeolite is used for the conversion of bulky molecules, such as heavy crude oil and biomass, this limitation becomes more evident. In such cases, hierarchical zeolites with intrinsic microporosities and integrated mesoporosities (and/or macroporosities) offer an effective solution to overcome the drawbacks of conventional microporous zeolites in catalytic applications [2–4]. To date, several synthesis strategies have been developed to produce hierarchical zeolites with multimodal pore architectures [5]. Most of these synthesis methods use mesoscale templates to create secondary porosity during the zeolite crystallisation process. Three classes of templating methodologies can be discerned: solid templating, supramolecular templating and indirect templating [5]. Considerable progress has been achieved in this field; nevertheless, this route is still far from being applied practically, because it is a complicated, expensive and environmentally unfriendly process. Moreover, the existing processes used to fabricate zeolites are not green at their sources, as they inevitably involve the use of synthetic aluminium- and silicon-containing chemicals. These chemicals are derived from natural aluminosilicate or silicate minerals through a series of complicated processes [6].

## MESOSCALE DEPOLYMERISATION-REORGANISATION STRATEGY

**Figure 1.**
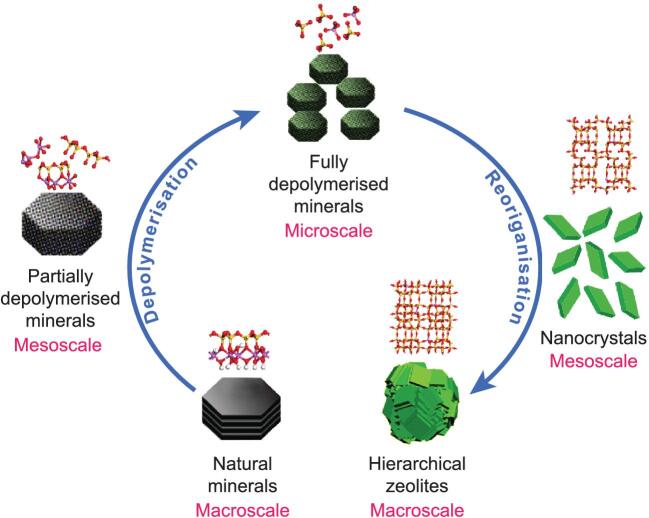
Schematic illustration of the proposed synthesis mechanism of the hierarchical zeolites via the mesoscale depolymerisation-reorganisation strategy.

To address the challenges above, a mesoscale depolymerisation-reorganisation strategy is proposed to directly synthesize hierarchical zeolites from natural aluminosilicate minerals, without using any mesoscale templates (see Fig. 1). This strategy consists of two basic steps: (i) top-down depolymerisation of minerals into zeolitic building units, including primary and secondary building units; and (ii) bottom-up reorganisation of the depolymerised minerals, which are composed of the rearrangement of zeolitic building units into zeolite nanocrystals. This is followed by the subsequent self-assembly of these nanocrystals into hierarchical zeolites.

The efficiency of mineral depolymerisation plays an important role in the synthesis of hierarchical zeolites, and thus much effort has been dedicated to the development of effective approaches. Thermal treatment at high temperatures (>600^o^C) is the traditional approach for the depolymerisation of minerals; however, the huge energy consumption required (ca. 459 kJ/mol kaolin at 600^o^C) largely limits its practical application. The activation of minerals can be achieved by the treatment of minerals under a sub-molten salt (SMS) medium. Furthermore, the attack of the lamellar structure of mineral by a high concentration of O^2−^ species in the SMS medium can lead to the decomposition of its crystalline structure at lower temperatures (∼250^o^C) in 2 hours [7]. However, the evaporation of a large amount of water during the SMS method results in additional energy consumption (1.25 MJ/kg kaolin) and waste water emissions (3.77 kg/kg kaolin). Moreover, this process is conducted in batch mode, and thus is unsuitable for large-scale industrial production. Recently, an industrially feasible quasi-solid-phase (QSP) depolymerisation approach has been developed. This method is achieved via the synergic effects between chemical and mechanical deconstructions, and thereby can be conducted in a continuous mode and under dramatically milder conditions [8]. Different characterization techniques that monitor the QSP depolymerisation process have revealed that the raw kaolin mineral undergoes a complete structural transformation. This leads to the conversion of its layer structure into porous particles, along with the formation of monomer orthosilicate anions and Al^IV^ species above 90^o^C in the activated product [8]. This structural transformation allows for the generation of highly active Si and Al precursors for the synthesis of zeolite. Moreover, QSP depolymerisation can be carried out in a continuous way, in an economical manner (low temperature of 100–250^o^C, low ratios of H_2_O/mineral and alkali/mineral and a short treatment time) [8]. Indeed, it has already been applied to the large-scale depolymerisation of minerals for zeolite synthesis.

Recent studies have proven that the bottom-up reorganisation of depolymerised mineral enables the successful synthesis of hierarchical zeolites with multiple-porosities [6–13]. It has also been confirmed that the formation of a hierarchical pore architecture involves the rearrangement of zeolitic building units and the self-assembly of zeolite nanocrystals [7]. The secondary building units of the zeolitic structure were detected in the depolymerised mineral, and this structure can act as ‘crystallization seeds’ to promote zeolite nucleation. This leads to a rapid rearrangement of the depolymerised mineral into zeolite nanocrystals. The formed zeolite nanocrystals are meta-stable, and their high surface free energy drives them to self-assemble into larger aggregates. This self-assembly of zeolite nanocrystals finally results in the synthesis of hierarchical zeolites. Both intra- and inter-crystalline mesopores are found in these hierarchical zeolites. The generation of intra-crystalline mesopores in hierarchical zeolites occurs due to the duplication of mesopores from the depolymerised mineral into the zeolite structure. Most of the intra-crystalline mesopores that exist in the depolymerised mineral are maintained during the bottom-up reorganisation process, and thus are inherited by the resulting hierarchical zeolites [7]. In contrast, the inter-crystalline mesopores come from the voids among the nanocrystals [7].

**Figure 2.**
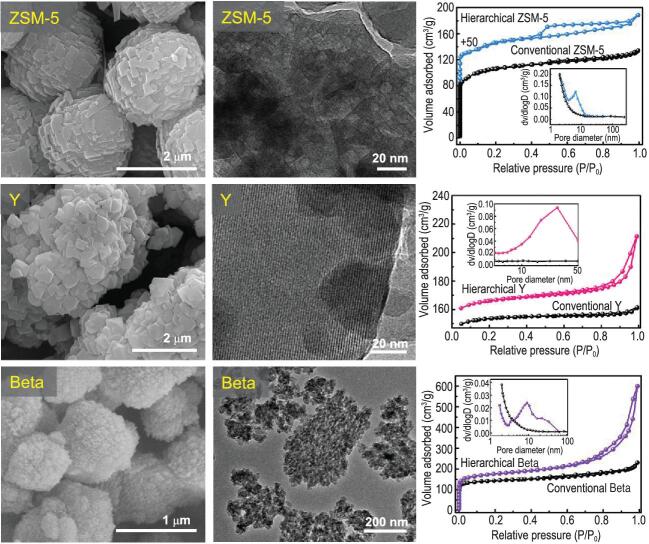
Scanning and transmission electron microscope images and N_2_ adsorption of hierarchical ZSM-5, NaY and Beta zeolites synthesized from natural minerals. This figure is adapted and reprinted with permission from [7–9].

In fact, this mesoscale depoly-merisation-reorganisation strategy has been proven as a general approach for the synthesis of hierarchical zeolites. Hierarchical zeolites with different topologies, such as ZSM-5, NaY and Beta, have been successfully fabricated using SMS/QSP depolymerised aluminium-rich kaolinite/rectorite and thermally activated silicon-rich diatomite as starting materials under this strategy [7–9]. Moreover, for the synthesis of Y zeolite, the atom economy (50.91%), energy consumption (35.688 MJ/tonne zeolite) and total waste emissions (1.961 tonne/tonne zeolite) of this synthesis strategy are much lower than those (32.79%, 46.259 MJ/tonne zeolite and 4.726 tonne/tonne zeolite) of the conventional synthesis route. As shown in Fig. 2, these industry relevant zeolites synthesised from depolymerised minerals exhibit two levels of porosity, consisting of intrinsic micropores and secondary mesopores. The intra-crystalline mesopores are visible in ZSM-5 and NaY; whereas inter-crystalline mesopores are found in Beta zeolite. The formation of mesopores in these zeolites without any template proves that bottom-up reorganisation is a green and economical method for the fabrication of hierarchical zeolites. Moreover, the *in situ* incorporation of impurity elements in the mineral into the hierarchical zeolite framework can also be realised. This results in the formation of hetero-atom containing zeolites (such as FeZSM-5 and TiZSM-5) [10,11]. The obtained hierarchical zeolites were studied in different catalytic reactions, including hydroisomerisation, aromatisation, esterification and the selective catalytic reduction of NO by NH_3_. In comparison to conventional zeolites, these hierarchical zeolites exhibited enhanced catalytic performance, due to the significantly alleviated diffusion limitation. Most importantly, the hierarchical ZSM-5 based catalyst has been successfully applied in the hydrotreatment of the fluid catalytic cracking naphtha in several industrial units (with a total capacity of approximately 10 million tonnes/a). This provides a key technical support for upgrading gasoline quality in China.

## FUTURE PROSPECTS

Previous studies have successfully developed a versatile mesoscale depolymerisation-reorganisation strategy to synthesize hierarchical zeolites from natural minerals. At this stage, the mechanisms of mesoscale depolymerisation and reorganisation are still not fully understood. Therefore, it is crucial to conduct *in situ*/operando studies (such as *in situ* resonance ultraviolet Raman, time-resolved X-ray synchrotron diffraction and *in situ* nuclear magnetic resonance) to track the synthesis process. This will allow an understanding of the depolymerisation-reorganisation mechanisms from the atomic level to the macro level. The knowledge gathered in these studies will guide the synthesis of hierarchical zeolites in a rational, controllable and sustainable way. In the near future, a practicable platform technology for the synthesis of hierarchical zeolites can be firmly established based on this mesoscale depolymerisation-reorganisation strategy.
